# Science Mapping: A Bibliometric Analysis on Cyberbullying and the Psychological Dimensions of the Self

**DOI:** 10.3390/ijerph20010209

**Published:** 2022-12-23

**Authors:** Ángel Denche-Zamorano, Sabina Barrios-Fernandez, Carmen Galán-Arroyo, Sebastián Sánchez-González, Felipe Montalva-Valenzuela, Antonio Castillo-Paredes, Jorge Rojo-Ramos, Pedro R. Olivares

**Affiliations:** 1 Promoting a Healthy Society Research Group (PHeSO), Faculty of Sport Sciences, University of Extremadura, 10003 Cáceres, Spain; 2 Faculty of Education, Psychology and Sport Sciences, Universidad de Huelva, 21007 Huelva, Spain; 3 Occupation, Participation, Sustainability and Quality of Life (Ability Research Group), Nursing and Occupational Therapy College, University of Extremadura, 10003 Cáceres, Spain; 4 Sección de Educación Física, Escuela de Suboficiales del Ejército de Chile, Maipú 9250000, Chile; 5 Laboratorio de Fisiología del Ejercicio y Metabolismo, Escuela de Kinesiología, Universidad Finis Terrae, Providencia 7501015, Chile; 6 Grupo AFySE, Investigación en Actividad Física y Salud Escolar, Escuela de Pedagogía en Educación Física, Facultad de Educación, Universidad de Las Américas, Santiago 8370040, Chile; 7 Physical Activity for Education, Performance and Health, Faculty of Sport Sciences, University of Extremadura, 10003 Caceres, Spain; 8 Facultad de Educación, Universidad Autonoma de Chile, Talca 3480094, Chile

**Keywords:** cyberbullying perpetration, cyberbullying victimization, self-concept, self-esteem, depression, cyberbullies, bystanders

## Abstract

Cyberbullying prevalence is increasing in the world, being a form of abuse that follows victims into their most intimate settings. Cyberbullying affects victims’ mental health, self-esteem, emotions, and academic performance. Cyberbullies present low levels of self-control and empathy. This research aimed to map scientific research on Cyberbullying and the Psychological Dimensions of the Self. A bibliometric analysis of scientific documents published in journals indexed in the Web of Science (WoS) was performed. Traditional bibliometric laws were applied and VOSviewer was used to generate visualizations. The annual publications followed exponential growth. *Computers in Human Behaviour* was the journal with the most publications. Researchers from the USA and Spain were the most prolific. Sameer Hinduja and Justin Patchin were the most cited authors. Hence, there is a growing interest among researchers in Cyberbullying and the emotional aspects of children and adolescents. The USA and Spain were the leading countries in research on this subject. Rosario Ortega-Ruiz, Sameer Hinduja and Justin Patchin were the most prolific and influential authors.

## 1. Introduction

### 1.1. Psychology of the Self

Although everyone seems to know what the term “self” means, defining it accurately is complex, having a variety of considerations [1]. However, it could be defined as a reference to personal identity, to the being and the experience of the individual as unique and separate from others, including his or her distinctive attributes, both conscious and unconscious, and to the physical and physiological [2]. The self encompasses several dimensions: physical, emotional, socio-cultural, mental and spiritual [3]. The physical self joins four aspects: physical qualities, physical capacity, skills, and the physical environment; the emotional dimension relates to feelings or qualities of affect and their management; the mental dimension deals with cognition, intelligence, ideas, thoughts, perceptions, will, mental formations and visions; the socio-cultural dimension is characterized by the understanding of the self and the awareness of the others’ self; and the spiritual one involves exploring the key principles, beliefs and values that give meaning and purpose to one’s life [1,3].

### 1.2. Bullying and Cyberbullying Conceptualisation

Bullying encompasses negative behaviours that one may receive from other peers in the school setting or in other environments [4] which can start at an early age or at any time during adolescence, and can be occasional or prolonged over time [5]. Thus, negative behaviours include hitting, threats, insults, false rumours, hurtful comments, and exclusion, among others [6], which can lead to vulnerability or defenselessness situations causing negative effects on their social relationships, mental health, self-concept [7], self-efficacy, emotions [8] physical health, and academic performance [9]. Cyberbullying is a form of bullying with high growth [10] resulting from the increase in new technology and new lifestyles [11] and is defined as “an intentional and repeated harm inflicted through the use of computers, mobile phones and other electronic devices” [12] with the aim of offending, harming, embarrassing and/or humiliating [13,14] peers who cannot defend themselves [15,16]. Therefore, Cyberbullying moves out of the school limits and pursues the victim into their intimate spaces through the virtual world [17,18], causing adverse effects on the physical and mental health of victims, even from the first episodes [19]. The prevalence of Cyberbullying is higher in males than in females, although the differences are narrowing [20]. Cyberbullying victims range from 10–50% of adolescents, with a perpetration prevalence of around 25% [21,22]. In Spain, cybervictimization and cyberperpetration prevalence average 25%, although prevalence ranges widely [23,24]. Therefore, Cyberbullying is a major concern for governments, social, health and educational systems [11,25,26,27,28], and for this reason, prevention and intervention programs are being implemented [29,30].

### 1.3. Cyberbullying Effects on the Self

Regarding different psychological dimensions, on the one hand, cybervictims suffer various negative effects such as increased depression and anxiety symptoms, and higher emotional stress [31,32]. Moreover, the self-concept dimensions, including self-image, self-esteem, and the ideal self, can be affected, as it is dynamic and malleable and can be influenced by social conditions [22,33]. Furthermore, the emotional dimensions can also be affected, as Cyberbullying causes low self-esteem, life dissatisfaction and low emotional self-efficacy. Loneliness and low self-concept are some of the most common effects and predictors among victims [34,35,36]. Usually, victims suffer from loneliness, lack of family and social support, and the absence of friends who can offer protection against the attacks and their consequences, so the socio-cultural aspects are also manifested [37]. On the other hand, cyberbullies also present high stress, anxiety, and life dissatisfaction, and this low self-control combined with poorer emotional empathy levels, are predictors for committing Cyberbullying [20,34,38]. Furthermore, having been a bullying victim may also be associated with an increased tendency to be a cyberbully [39,40], and having committed traditional bullying is also related to committing Cyberbullying at a later time [41]. Substantial differences were found between cybervictims and cyberbullies and gender concerning self-esteem, self-control, social and family support, and aggressiveness [37].

### 1.4. Cyberbullying and Bibliometric Analyses

Bibliometric studies allow a quantitative analysis of current scientific production on a specific topic, facilitating the calculation of general trends in publications, journals, keywords, researchers, and their institutions, locations, and networks, among others [42,43]. The resulting information is useful for finding authors, research groups and/or institutions or networks working on a subject to assist in the establishment of alliances and collaboration and to help researchers find journals or publishers interested in the topic [44]. In addition, they help to identify knowledge gaps and guide researchers to locate their contributions within a specific topic or area [45]. Furthermore, bibliometrics is objective, as it uses quantitative parameters of citation data from different studies through citation and co-citation analyses, counteracting the potential subjectivity of reviews [46]. Additionally, bibliometric studies are generating a lot of interest among researchers [47], since these studies allow for the identification of new research trends, favouring collaborations between researchers, and locating journals and articles of interest in a thematic area [48,49,50].

### 1.5. Study Aim

Given the existing connections between Cyberbullying and Psychological Dimensions of the Self, this work aims to establish the current status on this topic, assisting in the identification of new research areas or gaps in the knowledge on this subject. Hence, this study’s main objective was to conduct a scientific mapping of publications on Cyberbullying and the Psychological Dimensions of the Self, identifying the trend of annual publications, the most prolific and influential journals, authors and countries in the object of study, the most cited articles and the keywords most used by the authors, answering the following questions: what and how much research has been done regarding Cyberbullying and the Psychological Dimensions of the Self? Which are the most relevant journals related to this topic? Who are the most prolific and most cited authors? Which collaboration networks and countries or regions are the most relevant? What are the most used keywords?

## 2. Materials and Methods

### 2.1. Search Strategy and Data Sources

The scientific mapping was based on research on Cyberbullying and terms related to the psychology of the self: self-concept, self-esteem, self-efficacy, self-knowledge, self-image, self-control, self-worth, self-construction, self-identity, self-perspective, self-structure, social self and the ideal self according to data extracted from the Web of Science (WoS) Core Collection database of Clarivate Analytics, in its Science Citation Index Expanded (SCI-Expanded), Social Sciences Citation Index (SSCI) and Emerging Sources Citation Index (ESCI). The WoS was chosen as a data source because it is one of the most prestigious and most used data sources in the scientific field for bibliometric analysis, collecting the publications of the most relevant journals, and because it allows the export of complete information on scientific publications (authors, title, abstract, keywords, sources, publisher information, affiliations, country/regions, cited references, document types, funding information, etc.) for subsequent analysis [47,51]. This is the reason why WoS is one of the most widely used data sources for bibliometric analysis [52].

An advanced search was carried out in WoS, using the following search vector: (TI = (“cyberbullying”) or AK = (cyberbullying)) and (TS = (“self-concept”) or TS = (“self-esteem”) or TS = (“self-efficacy”) or TS = (“self-know*”) or TS = (“social self”) or TS = (“self-image”) or TS = (“self-control”) or TS = (“self-worth”) or TS = (“ideal self”) or TS = (“self-construction”) or TS = (“self-identity”) or TS = (“self-perspective”) or TS = (“self-structure”)). In WoS, the tag “TI” allows searches for articles with a specific term in the title, “AK” is used for searches for articles by author keywords; and “TS” is used for searches for articles with specific words in the topic (Title, Abstract, Author keywords and Keyword Plus^®^). The search was conducted on 17 July 2022, limiting the results to articles and reviews of articles, with no time or language restrictions. The search resulted in a total of 329 documents (308 articles and 21 reviews). The data were extracted in .xslx format to be analyzed with Microsoft Excel for Microsoft 365 MSO version 2206 (Microsoft, Redmond, WA, USA), and in plain text, to be analysed with VoSViewer (Leiden University, Leiden, The Netherlands), as this software allows the creation of bibliometric maps in an easy-to-interpret way [53].

### 2.2. Data Analysis

A descriptive analysis of the annual publications on the topic was conducted using the WoS analyze reports tool. The trend followed by the annual publications was analyzed, checking whether these were in a phase of exponential growth, applying De Solla Price’s law of exponential growth of science [54,55], and evaluated with the coefficient of determination (R^2^) adjusted to an exponential growth ratio. Only years with complete data were considered for this analysis. The WoS Analyze Reports tool was also used to provide a descriptive analysis of the WoS thematic categories to which the publications were related. Bradford’s law of concentration of science was applied to identify the journals with the highest number of publications on the topic, and those that accumulated the highest number of citations in the articles published on the topic [56]. Lotka’s law was used to identify the most prolific authors in the field of study [57], and the Hirsch index (h-index) was applied to these authors, considering most prominent the most prolific authors who presented n papers on the subject with n citations [58]. Zipf’s law was used to highlight the keywords most used by the authors in the articles analyzed [59]. Thus, to identify the most cited documents among those analyzed, the h-index was used, considering the n documents with n citations as the most relevant articles on the topic [60]. Similarly, the h-index was used to identify the references most used by the authors. VoSviewer software, with the strength of association and fractionalization analysis, was used to create visualizations of the interrelationships between journals, authors, countries/regions, and keywords.

## 3. Results

### 3.1. Exponential Growth

**Figure 1:**
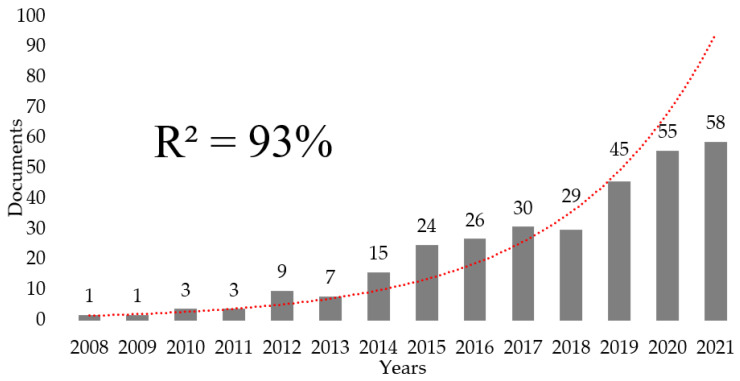
Graph showing the trend of annual publications on the topic.

The 329 publications found were published between 2008 and 2022. The annual publications followed an exponential growth between 2008 and 2021 (2022 was excluded as it did not present complete data on annual publications), with R^2^ adjusted at 90% to an exponential growth rate (Figure 1).

### 3.2. WoS Categories

Documents were classified into 48 WoS thematic categories. Table 1 shows the categories with the most publications linked and the journals and publishers with the most publications in each category. The category with the highest number of documents was Psychology Multidisciplinary (83), with Computers in Human Behaviour (32) and Elsevier (39) being the most prolific journal and publisher in this category, respectively.

### 3.3. Publications Titles

The papers were published in 158 journals. Based on the number of publications, Bradford’s Core was formed by the 10 most prolific journals on the topic (Table S1). The distribution presented by the Core (10), Zone I (36) and Zone II (112) journals according to the number of documents conformed to Bradford’s theoretical series with an error rate of 1.6% (Table S2). These journals contributed 34.3% of the publications. *Computers in Human Behavior* (Pergamon-Elsevier) was the journal with the most publications (32). According to the number of citations, there were four core journals: *Journal of Adolescent Health* (Elsevier), *Computers in Human Behavior* (Pergamon-Elsevier), *Archives of Suicide Research* (Routledge-Taylor & Francis) and *Aggression and Violent Behavior* (Pergamon-Elsevier), comprising 36% of the total number of citations. Table 2, Bradford’s Core and Zone I journals by the number of citations, including four journals in the Core, 14 in Zone I and 140 in Zone II according to the number of citations which conformed to Bradford’s theoretical series with an error rate of −35% (Table S2). Except for *Emotional and Behavioural Difficulties* (Routledge-Taylor & Francis) indexed in ESCI, all Bradford’s Core and Zone I journals had a Journal Impact Factor (JIF), eight of them in the first quartile (Q1), six in the second (Q2) and three in the third (Q3).

### 3.4. Most Prolific and Influential Co-Authors

After conducting the co-authorship analysis, 837 co-authors were found. More than 80% of co-authors had published a unique paper (679 co-authors), 16% of co-authors published between two and four papers (135 co-authors), and only 3% (23 co-authors) published five or more papers. These last ones were considered the most prolific co-authors, given that the application of Lotka’s law estimated a number lower than 29 (square root of 837). Rosario Ortega-Ruiz (11 papers) was highlighted as the most prolific co-author. Applying the h-index to the 23 most prolific co-authors, 22 co-authors with at least 22 citations within the dataset were highlighted as the most influential co-authors (Table 3). From these, only Pencheng Wang (five papers and 20 citations) was not included among the most influential. The most influential co-authors were Sameer Induja and Justin Patchin (six papers and 1587 citations), Rosario Ortega-Ruiz (11 papers and 521 citations), and Hen Chang and Dennis Wong (five papers and 332 citations).

**Figure 2:**
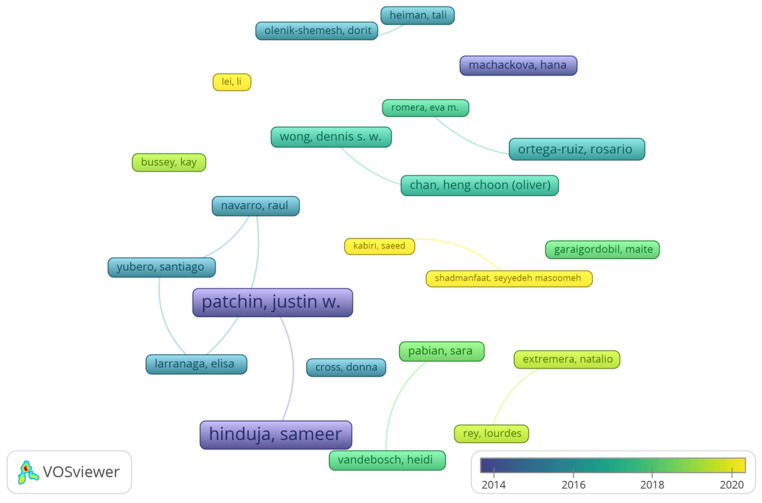
Graph with the most relevant co-authors: size node (citations) and colour (average publication years).

Figure 2 (graph fractionalization analysis: attraction: 10, repulsion: −2, node size: citations number, colour: average years of publication) and Figure S1 (graph with fractionalization analysis: attraction: 10, repulsion: −2, node size: number of papers, colour: average citations) display the 22 most influential co-authors and their interrelationships.

### 3.5. Countries/Regions

**Figure 3:**
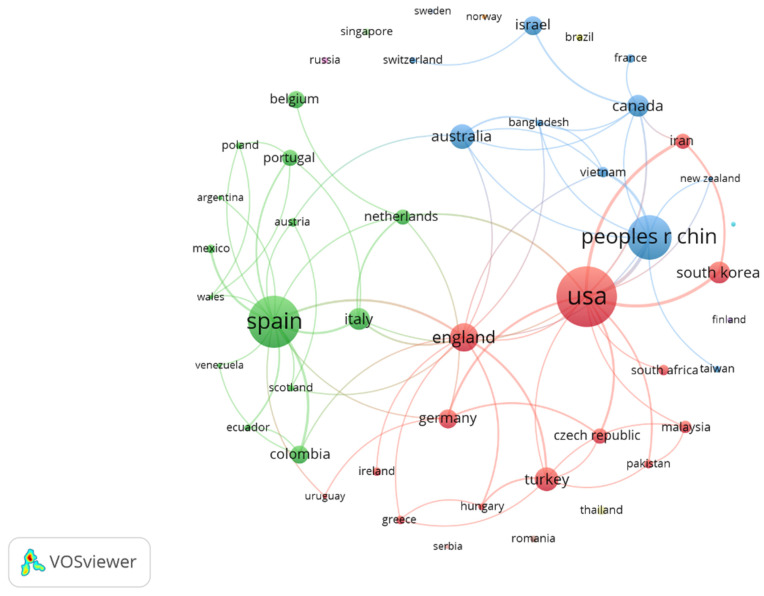
Graph with co-authored countries/regions: clusters formed by countries/regions; node size (documents).

The USA (80 documents), Spain (62 documents) and China (46 documents) were the three most prolific countries/regions among the 48 co-authored countries/regions found, leading each of the three most important publication clusters. By the number of citations, the USA (3678), Spain (1419) and England (1003) were the most influential countries/regions. The USA led the most prolific cluster, publishing with countries/regions such as England, South Korea, Turkey, Iran, and Germany. China led other important publishing clusters, publishing with Australia, Vietnam, Canada, and Israel. Meanwhile, Spain led a publication cluster with countries/regions such as Italy, the Netherlands, Portugal, Belgium, and many Spanish-speaking countries (Mexico, Colombia, Ecuador, etc.), and it was the country/region with the highest number of collaborations with other countries/regions (15), followed by the USA (14) and England (13). Figure 3 (graph with fractionalization analysis: attraction: 8; repulsion: 0; clustering resolution: 0.5; colour: cluster; size node: documents) shows the clusters formed by the countries/regions; while Figure S2 shows the average number of citations for every country/region (graph with fractionalization analysis: attraction: 8; repulsion: 0; clustering resolution: 0.5; colour: average of citations; size node: documents).

### 3.6. Documents

They were found a total of 48 documents with at least 48 citations in the WoS core, considering these as the most cited articles (Table S3). Figure S3 shows the graph with the 48 most cited papers and the citations among them (graph with fractionalization analysis: attraction: 10, repulsion: 0, node size: links, colour: citations). The most cited article was *Bullying, Cyberbullying and Suicide* [12], a study article by Sameer Hinduja and Justin Patchin published in 2010 with 835 citations. Among the top 10 most cited articles, the average year of publication was 2012, with *Cyberbullying, self-esteem, empathy and loneliness* [34] being the most recent article (2015) in the tenth position with 159 citations. Moreover, the 180-day usage count is a measure of the current level of interest in an article in WoS, based on the number of links clicks to the full article on the publisher’s website or the number of downloads [61]. However, none of the top 10 most cited articles was among the top five most used documents in the last 180 days in WoS. These five papers were: *A meta-analysis of factors predicting cyberbullying perpetration and victimization: From the social cognitive and media effects approach* (62 usages) [25], *Traditional school bullying and cyberbullying in Chinese societies: Prevalence and a review of the whole-school intervention approach* (58 usages) [62], *Bystander responses to cyberbullying: the role of perceived severity, publicity, anonymity, type of cyberbullying, and victim response* (48 usages) [63], *Cyberbullying in elementary and middle school students: a systematic review* (45 usages) [64] and *Standing up or standing by understanding bystanders’ proactive reporting responses to social media harassment* (41 usages) [65]. From the papers published between 2015 and 2022, none were among the top 10 most cited of all time. In the last 180 days, the articles in the top 10 received between three and 34 citations, with the most cited being *Psychological, physical, and academic correlates of Cyberbullying and traditional bullying* (34 usages) [41].

### 3.7. Author Keywords

**Figure 4:**
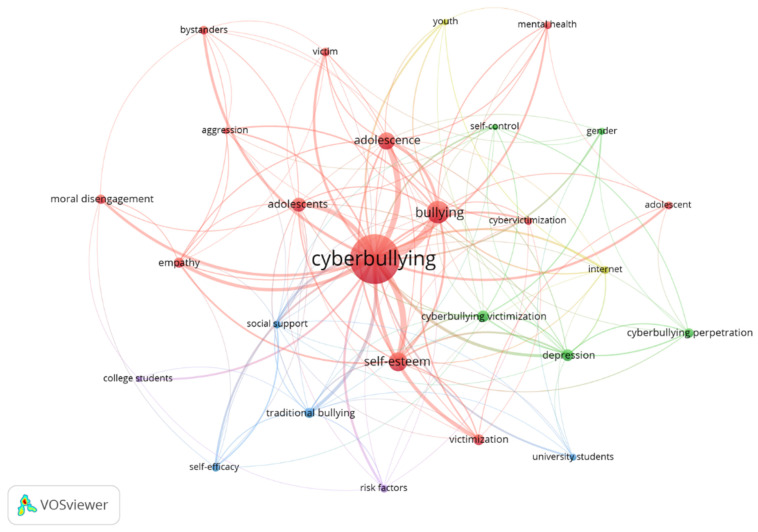
Keywords most used by the co-authors.

A total of 778 concepts were found in the co-occurrence analysis of keywords used by the authors. After applying Zipf’s law, it was estimated that the most frequent keywords should be a number equal to or less than 28 (square root of 778). Thirty-six words were found with six or more occurrences, and 27 were found with seven or more. These last ones were taken as those of most interest to the authors. Figure 4 shows the 27 keywords most used by the authors (graph with fractionalization analysis: attraction: 6, repulsion: −2, size node: number of occurrences, colour: cluster). The terms “cyberbullying” (233 occurrences) and “bullying” (58 occurrences), terms such as “self-esteem” (44), “adolescents” and “adolescence” (37 and 26, respectively), and “depression” (19) were the most used. Concepts such as Cyberbullying victimization, Cyberbullying perpetration, moral disengagement, or university students presented the most recent mean years of publication, being terms of current interest (Figure S4).

## 4. Discussion

### 4.1. Main Findings and Theoretical Implications

Although there are other bibliometric analyses on Cyberbullying [47,51,66], this research is the first to elaborate a scientific mapping of Cyberbullying and Psychological Dimensions of the Self on publications from journals indexed in the WoS, providing valuable knowledge on the trend of annual publications, journals and co-authors most interested and most influential on the topic, the most productive countries/regions, articles over time and in recent dates, and the concepts of greatest interest to the authors.

The first relevant finding was the exponential growth followed by annual publications from 2008 to 2021, which indicates the existence of a broad base of researchers, journals, and institutions with an interest in developing the subject and making such research relevant. Other studies had shown increasing interest in Cyberbullying in the last decade [47,67], with a decrease in publications in recent years [47]. Regarding publications on Cyberbullying and the psychological dimensions of self, there was also an important growth in 2014 and the following years, although a greater interest has been raised during the years 2019–2021.

Cyberbullying and Psychological Dimensions of the Self are topics of great interest for researchers from different areas, although they are mainly addressed by the fields of psychology and education. Educators, teachers, social workers, pedagogues, and psychologists are the most interested agents in the research topic, concerned with the effects of Cyberbullying on psychological dimensions including self-concept, self-esteem, self-confidence, self-efficacy, self-image, and self-control. However, more research is needed in education, on the effects of Cyberbullying on students’ emotions, academic performance, and the classroom environment, and for subsequent implementation as prevention and intervention programmes in scholarly settings. Moreover, among all the retrieved reviews, only three were related to educational categories. Continuing with the reviews, Kimberly L. Mason (2008) [68] published one of the most cited articles (149 citations), providing an overview of Cyberbullying, explaining how it differs from traditional bullying, highlighting the psychological impact on the bullied, and providing guidelines for the school staff. Hence, recent reviews have followed this line researching the need for school-based prevention, early detection, and intervention programs.

*Computers in Human Behaviours* (32 documents and 937 citations) and *The Journal of Adolescent Health* (three documents and 1017 citations) were the most prolific and cited journals (being either multidisciplinary or belonging to experimental or developmental psychology). Moreover, *The International Journal of Environmental Research and Public Health* (14 documents) and *Frontiers in Psychology* (12 documents), both multidisciplinary open access journals, were among the most prolific. Moreover, *The Archives of Suicide Research* (835 citations) was another of the most cited journals, thanks to Hinduja and Patchin’s manuscript, *Bullying, Cyberbullying and Suicide* [10], the most cited article in the dataset. Thus, no educational journals were found among the most prolific and most cited, but some that were interested in the topic were *The Journal of School Violence, Psychology in the School* and *The Journal of School Health* [47].

The countries most interested in the topic were the USA and Spain. The research group formed by Hinduja and Patchin (USA) was the most important in the field, followed by the cluster formed by Ortega-Ruiz and Romera, Larrañaga, Navarro and Yubero, Rey and Extremera, and Garaigordobil (Spain). These findings are in line with other bibliometrics on Cyberbullying [47,51]. Nevertheless, other countries are becoming involved in the topic, forming collaborations between authors from different countries, given that it is a worldwide issue. A variety of topics was found among the most cited articles. The most cited article, *Bullying, Cyberbullying and Suicide* [12] deals with suicidal thoughts in young people who have suffered bullying and Cyberbullying, suggesting that bullying situations should be considered a serious problem by educational agents and families. Cyberbullying and suicide represent a concern for researchers given the presence of other papers among the most cited [69,70]. Cyberbullying affects mental health, empathy, risk factors, self-esteem, self-efficacy, self-control, support from parents and friends, and the need for prevention and intervention programs are important topics given the papers most cited by researchers [32,41,71,72,73,74] and author keywords. Additionally, concepts such as Cyberbullying perpetration, victimization or the role of bystanders seem to generate greater interest in the most recent publications [25,62,63]. Those results are in line with the increasing interest in this issue [75,76].

### 4.2. Practical Applications

Cyberbullying and its psychological consequences have attracted researchers’ attention, as it is related to all psychological dimensions, comprehensively impacting the people who suffer from it. Prevention and intervention programs against Cyberbullying should include the Psychological Dimensions of the Self (emotional intelligence, self-control, self-confidence, empathy, self-efficacy, and emotional and social aspects [29,77]. Some interventions have demonstrated their effectiveness. For example, increasing confidence and self-efficacy seems to prepare children and adolescents to better cope with Cyberbullying [78]. Similarly, defensive self-efficacy and empathic self-efficacy increasingly seem to favour bystanders’ defence and denunciation of these abusive behaviours [78]. Furthermore, moral disaffection and identity, empathy, anger traits or school climate are predictors of Cyberbullying that need to be identified by educational stakeholders [64,79].

As illustrated throughout this paper, there is a growing interest in this subject of study (90% exponential growth rate). According to the bibliometrics laws, a topic has several stages of development: the precursor stage, then a phase of exponential growth, then linear growth, and finally a collapse (few articles are produced) or an alternative revival (when a few articles are produced) can happen [54]. Moreover, the most important countries/regions, collaborative networks, authors, journals, papers, author keywords and research topics of interest were highlighted. This data can support the collaboration between researchers, publishers, and journals, facilitating the location of experts and papers in the field, as well as journals potentially interested in the manuscripts derived from this topic.

Despite researchers’ and journals’ interests in this subject, our findings indicate that certain psychological dimensions are more studied than others. Thus, self-esteem, self-efficacy and self-control have been the most researched aspects, while future research should consider aspects such as self-image, self-esteem and body image to further explore Cyberbullying consequences.

### 4.3. Limitations and Future Lines

Although the reference database for many authors and the most widely used in the bibliometric analysis, the WoS, was used, this may have excluded documents published in journals that were not indexed in it, generating a bias when analyzing the field of study. Future research with a different design would be appropriate. Systematic reviews and meta-analyses could complement this research, and other research focusing on the aspects recognised in this research, such as the effects of prevention programmes, detection and intervention, and profiles prevalences, among others.

## 5. Conclusions

Research on Cyberbullying experienced exponential growth between 2008 and 2021, generating great interest among researchers and publishers. The most prolific journal was *Computers in Human Behavior,* while the most cited was *The Journal of Adolescent Health*. Rosario Ortega-Ruiz was the most prolific author, while Sameer Induja and Justin Patchin were the most cited. The countries most interested in the topic were the USA and Spain, as they had the highest number of publications and the more powerful relations with other countries. The most cited article was *Bullying, Cyberbullying and Suicide,* and the author’s keywords of greatest interest beyond Cyberbullying and Bullying were “self-esteem”, “depression”, and terms related to adolescence.

## Figures and Tables

**Table 1:** Top 10 Web of Science (WoS) thematic categories according to the number of publications.

WoS Categories (Documents)	Main Journal (Documents)	Main Publisher (Documents)
Psychology Multidisciplinary (83)	*Computers in Human Behavior* (32)	Elsevier (39)
Criminology Penology (53)	*Journal of Interpersonal Violence* (12)	Sage (22)
Family Studies (34)	*Journal of Interpersonal Violence* (12)	Sage (15)
Psychology Experimental (33)	*Computers in Human Behavior* (32)	Elsevier (33)
Psychology Developmental (31)	*Journal of Adolescence* (5)	Taylor & Francis (8)
Education Educational Research (27)	*Journal of School Violence* (4)	Taylor & Francis (8)
Psychiatry (21)	*Journal of Affective Disorders* (5)	Elsevier (8)
Public Environmental Occupational Health (27)	*International Journal of Environmental Research and Public Health* (15)	MDPI (15)
Social Work (23)	*Children and Youth Services Review* (10)	Elsevier (12)
Psychology Educational (21)	*Journal of School Violence* (4), *Psychology of Schools* (4)	Taylor & Francis (5)
Psychology Social (21)	*Deviant Behavior* (7)	Taylor & Francis (7)

**Table 2:** Bradford’s Core and Zone I journals according to the number of citations.

Bradford’s Zone	Journals (Publishers)	NºDoc.	Cit.	%Cit.	JIF	Q.	%O.A.
CORE	*Journal of Adolescent Health* (Elsevier)	3	1017	11	7.830	Q1	14.9
	*Computers in Human Behavior* (Pergamon-Elsevier)	32	937	10.1	8.957	Q1	11.3
	*Archives of Suicide Research* (Routledge-Taylor & Francis)	1	835	9	2.833	Q2	7.1
	*Aggression and Violent Behavior* (Pergamon-Elsevier)	6	556	6	4.874	Q1	4.3
ZONE I	*Journal of School Health* (Wiley)	2	397	4.3	2.460	Q2	7.5
	*Aggressive Behavior* (Wiley)	6	393	4.2	3.047	Q2	17.7
	*Youth & Society* (Sage Publications)	4	238	2.6	2.793	Q2	6
	*Frontiers in Psychology* (Frontiers Media)	12	216	2.3	4.232	Q1	99.3
	*Journal of Child Psychology and Psychiatry* (Wiley)	1	200	2.2	8.265	Q1	32.8
	*Journal of Community & Applied Social Psychology* (Wiley)	2	199	2.1	2.968	Q3	23.4
	*Journal of Affective Disorders* (Elsevier)	5	186	2	6.533	Q1	10
	*Journal of Youth and Adolescence* (Springer)	3	183	2	5.625	Q1	22.3
	*Children and Youth Services Review* (Pergamon-Elsevier)	10	178	1.9	2.519	Q1	8.7
	*Emotional and Behavioural Difficulties* (Routledge-Taylor & Francis)	3	177	1.9	n.a.	n.a.	16.5
	*Psychology in the Schools* (Wiley)	4	176	1.9	1.923	Q3	4.7
	*Developmental Neurorehabilitation* (Taylor & Francis)	1	175	1.9	1.907	Q3	14.7
	*Journal of Interpersonal Violence* (Sage Publications)	12	170	1.8	2.621	Q2	4.5
	*New Media & Society* (Sage Publications)	4	150	1.6	5.310	Q1	24.8

Nº Doc. (Number of documents); Cit. (Number of citations); % Cit. (Percentage of citations); JIF (Journal impact factor); % O.A. (Percentage of open access); Q. (JIF Quartile); n.a. (not applicable).

**Table 3:** Most prolific and prominent co-authors on Cyberbullying and Psychology of the Self.

Co-Authors	Affiliation/Countries-Regions	Documents	Citations
Ortega-Ruiz, R.	University of Cordoba/Spain	11	521
Garaigordobil, M.	University of the Basque Country/Spain	7	119
Kabiri, S.	University of Mazandaran/Iran	7	37
Pabian, S.	University of Antwerp/Belgium	7	195
Shadmanfaat, S.M.	University of Guilan/Iran	7	37
Vandebosch, H.	University of Antwerp/Belgium	7	222
Bussey, K.	Macquarie University/Australia	6	158
Hinduja, S.	Florida Atlantic University/EEUU	6	1587
Larranaga, E.	University of Castilla-La Mancha/Spain	6	198
Lei, L.	Renmin University of China/China	6	48
Navarro, R.	University of Castilla-La Mancha/Spain	6	198
Patchin, J.W.	University of Wisconsin System/EEUU	6	1587
Romera, E.M.	University of Cordoba/Spain	6	83
Yubero, S.	University of Castilla-La Mancha/Spain	6	198
Chan, H.C.	City University of Hong Kong/China	5	332
Cross, D.	University of Western Australia/Australia	5	123
Extremera, N.	University of Malaga/Spain	5	110
Heiman, T.	Open University Israel/Israel	5	152
Machackova, H.	Masaryk University/Czech Republic	5	259
Olenik-Shemesh, D.	Open University Israel/Israel	5	152
Rey, L.	University of Malaga/Spain	5	110
Wong, D.S.	City University of Hong Kong/China	5	332

## Data Availability

Datasets are available through the corresponding author upon reasonable request.

## Supplementary Materials

The following supporting information can be downloaded at: https://www.mdpi.com/article/10.3390/ijerph20010209/s1, Figure S1: Graph with most influential co-authors: side node (documents) and colour (average of citations); Figure S2: Chart with co-authoring countries_regions; Figure S3: Graph with 48 documents most cited and links betweens them; Figure S4: Keywords; Table S1: Core and bradford zone 1 journals; Table S2: Bradford’s zones; Table S3: Documents more cited 48 documents with 48 or more cites.
