# Supplementary material for: Science Mapping: A Bibliometric Analysis on Cyberbullying and the Psychological Dimensions of the Self
Source: Int J Environ Res Public Health. 2022 Dec 23;20(1):209. doi: 10.3390/ijerph20010209 (PMC9819207; doi:10.3390/ijerph20010209)
Supplement: Supplementary file 1 [file ijerph-20-00209-s001.zip › Table S1. Core and bradford zone 1 journals.pdf]

Table S1. Bradford's Core and Zone I journals according to number of documents.

Bradford's zone	Journals (Publishers)	Nº Doc.	% Doc.	Nº Cit.	JIF	Q.	% O.A.
CORE	Computers in Human Behavior (Pergamon-Elsevier)	32	11.0%	937	8.957	Q1	11.3%
	International Journal of Environmental Research and Public Health (MDPI)	14	10.1%	119	4.614	Q1	95.0%
	Frontiers in Psychology (Frontiers Media)	12	9.0%	216	4.232	Q1	99.5%
	Journal of Interpersonal Violence (Sage Publications)	12	6.0%	170	2.621	Q2	4.7%
	Children and Youth Services Review (Pergamon-Elsevier)	10	4.3%	178	2.519	Q1	8.7%
	Deviant Behavior (Taylor & Francis)	7	4.2%	85	1.716	Q3	5.7%
	Crime & Delinquency (Sage Publications)	7	2.6%	70	2.307	Q2	6.3%
	Current Psychology (Springer)	7	2.3%	37	2.387	Q2	15.7%
	Aggression and Violent Behavior (Pergamon-Elsevier)	6	2.2%	556	4.874	Q1	4.3%
	Aggressive Behavior (Wiley)	6	2.1%	393	3.047	Q2	17.7%

Nº Doc. (Number of documents); Nº Cit. (Number of citations); % Doc. (Percentage of documents); JIF (Journal Impact Factor); % O.A. (Percentage of open access); Q. (JIF Quartile); n.a. (not applicable).
