# Supplementary material for: Science Mapping: A Bibliometric Analysis on Cyberbullying and the Psychological Dimensions of the Self
Source: Int J Environ Res Public Health. 2022 Dec 23;20(1):209. doi: 10.3390/ijerph20010209 (PMC9819207; doi:10.3390/ijerph20010209)
Supplement: Supplementary file 1 [file ijerph-20-00209-s001.zip › Table S2. Bradford's zones.pdf]

Table S2. Bradford's zones and their number of journals, according to number of documents and citations.

According to number of documents												
Zone	Nº journals (%)		Number of documents (%)		Acc. nº journals (%)		Acc. nº documents (%)		Bradford multipliers		Journals (theoretical serie)	
CORE	10	(6.3%)	113	(34.3%)	10	(6.3%)	113	(34.3%)			n0	10
Zone 1	36	(22.8%)	104	(31.6%)	46	(29.1%)	217	(66.0%)		3.60	n1	34
Zone 2	112	(70.9%)	112	(34.0%)	158	(100%)	329	(100%)		3.11	n2	113
Total	158	100%	329	100%					Mean	3.36		156
											% Error	1.2%

According to number of citations												
Zone	Nº journals (%)		Number of citatios (%)		Acc. nº journals (%)		Acc. nº citations (%)		Bradford multipliers		Journals (theoretical serie)	
CORE	4	(2.5%)	3345	(36.0%)	4	(2.5%)	3345	(36.0%)			n0	4
Zone 1	14	(8.9%)	3038	(32.7%)	18	(11.4%)	6383	(68.7%)		3.50	n1	27
Zone 2	140	(88.6%)	2896	(31.2%)	158	(100%)	9279	(100%)		10.00	n2	182
Total	158	100%	9279	100%					Mean	6.75		213
											% Error	-35.0%

Nº (Number); % (Percentage); Acc (Accumulated).
