# Supplementary material for: Science Mapping: A Bibliometric Analysis on Cyberbullying and the Psychological Dimensions of the Self
Source: Int J Environ Res Public Health. 2022 Dec 23;20(1):209. doi: 10.3390/ijerph20010209 (PMC9819207; doi:10.3390/ijerph20010209)
Supplement: Supplementary file 1 [file ijerph-20-00209-s001.zip › Table S3. Documents more cited 48 documents with 48 or more cites.pdf]

Table S3. Documents more cited: 48 documents with 48 or more cites.

Title. Main author (Year of publication)	Journal Abbreviation	Cites
Bullying, Cyberbullying, and Suicide. Hinduja, S. (2010)	Arch Suicide Res	835
Psychological, Physical, and Academic Correlates of Cyberbullying and Traditional Bullying. Kowalski, R. (2013)	J Adolescent Health	541
Online Communication Among Adolescents: An Integrated Model of Its Attraction, Opportunities, and Risks. Valkenburg, P. (2011)	J Adolescent Health	458
Cyberbullying and Self-Esteem. Patchin, J. (2010)	J School Health	299
Annual Research Review: Harms experienced by child users of online and mobile technologies: the nature, prevalence and management of sexual and aggressive risks in the digital age. Livingstone, S. (2014)	J Child Psychol Psyc	200
Traditional and Nontraditional Bullying Among Youth: A Test of General Strain Theory. Patchin, J. (2011)	Youth Soc	198
The Emotional Impact of Bullying and Cyberbullying on Victims: A European Cross-National Study. Ortega-Ruiz, R. (2012)	Aggressive Behav	184
Cyberbullying among students with intellectual and developmental disability in special education settings. Didden, R. (2009)	Dev Neurorehabil	175
Social Influences on Cyberbullying Behaviors Among Middle and High School Students. Hinduja, S. (2013)	J Youth Adolescence	173
Cyberbullying, self-esteem, empathy and loneliness. Brewer, G. (2015)	Comput Hum Behav	159
Cyberbullying: A preliminary assessment for school personnel. Mason, K. (2008)	Psychol Schools	149
Systematic review of theoretical studies on bullying and cyberbullying: Facts, knowledge, prevention, and intervention. Zych, I. (2015)	Aggress Violent Beh	147
Traditional school bullying and cyberbullying in Chinese societies: Prevalence and a review of the whole-school intervention approach. Chan, H. (2015)	Aggress Violent Beh	141
A meta-analysis of factors predicting cyberbullying perpetration and victimization: From the social cognitive and media effects approach. Chen, L. (2017)	New Media Soc	139
Longitudinal Risk Factors for Cyberbullying in Adolescence. Sticca, F. (2013)	J Community Appl Soc	124
Cyberbullying perpetration and victimization among adolescents in Hong Kong. Wong, D. (2014)	Child Youth Serv Rev	116
Are cyberbullying intervention and prevention programs effective? A systematic and meta-analytical review. Gaffney, H. (2019)	Aggress Violent Beh	111
The Role of Moral Disengagement and Self-Efficacy in Cyberbullying. Bussey, K. (2015)	J Sch Violence	110
Personal Characteristics and Contextual Factors That Determine Helping, Joining In, and Doing Nothing When Witnessing Cyberbullying. Van Cleemput, K. (2014)	Aggressive Behav	107
Cyberbullying in context: Direct and indirect effects by low self-control across 25 European countries. Vazsonyi, A. (2012)	Eur J Dev Psychol	101
Relationships Among Cyberbullying, School Bullying, and Mental Health in Taiwanese Adolescents. Chang, F. (2013)	J School Health	98
Does the offline bully-victimization influence cyberbullying behavior among youths? Application of General Strain Theory. Jang, H. (2014)	Comput Hum Behav	91
Parenting in the digital era: Protective and risk parenting styles for traditional bullying and cyberbullying victimization. Martinez, I. (2019)	Comput Hum Behav	88
Am I at risk of cyberbullying? A narrative review and conceptual framework for research on risk of cyberbullying and cybervictimization: The risk and needs assessment approach. Baldry, A. (2015)	Aggress Violent Beh	85
Cultivating youth resilience to prevent bullying and cyberbullying victimization. Hinduja, S. (2017)	Child Abuse Neglect	82
Predictors of victimisation across direct bullying, indirect bullying and cyberbullying. Brighi, A. (2012)	Emot Behav Diffic	81
The Influence of School Climate and Family Climate among Adolescents Victims of Cyberbullying. Ortega-Baron, J. (2016)	Comunicar	80
From cyberbullying to electronic aggression: typology of the phenomenon. Pyzalski, J. (2012)	Emot Behav Diffic	80
Cyberbullying Perpetration and Victimization Among Middle-School Students. Rice, E. (2015)	Am J Public Health	77

Bystanders' Support of Cyberbullied Schoolmates. Machackova, H. (2013)	J Community Appl Soc	75
Cyberbullying and LGBTQ Youth: A Systematic Literature Review and Recommendations for Prevention and Intervention. Abreu, R. (2018)	J Child Adoles Traum	74
Cyberbullying, psychological distress and self-esteem among youth in Quebec schools. Cenat, J. (2014)	J Affect Disorders	72
Problematic Online Behaviors among Adolescents and Emerging Adults: Associations between Cyberbullying Perpetration, Problematic Social Media Use, and Psychosocial Factors. Kircaburun, K. (2019)	Int J Ment h¡Health Ad	70
Unique effects of different components of trait emotional intelligence in traditional bullying and cyberbullying. Baroncelli, A. (2014)	J Adolescence	70
Longitudinal impact of the Cyber Friendly Schools program on adolescents' cyberbullying behavior. Cross, D. (2016)	Aggressive Behav	69
Cyberbullying: a storm in a teacup? Wolke, D. (2017)	Eur Child Adoles Psy	67
Cyberbullying Victimization, Self-Esteem and Suicidal Ideation in Adolescence: Does Emotional Intelligence Play a Buffering Role? Extremera, N. (2018)	Front Psychol	63
Children's Cyberbullying Victimization: Associations with Social Anxiety and Social Competence in a Spanish Sample. Navarro, R. (2012)	Child Indic Res	61
Cyberbullying and self-esteem: An Italian study. Palermiti, A. (2017)	Comput Hum Behav	60
Adolescent victims of cyberbullying: prevalence and characteristics. Estevez, A. (2010)	Behav Psychol	58
Cyberbullying involvement among students with ADHD: relation to loneliness, self-efficacy and social support. Heiman, T. (2015)	Eur J Spec Needs Edu	58
Longitudinal and Incremental Relation of Cybervictimization to Negative Self-Cognitions and Depressive Symptoms in Young Adolescents. Cole, D. (2016)	J Abnorm Child Psych	57
The process of coping with cyberbullying: A systematic review. Raskauskas, J. (2015)	Aggress Violent Beh	55
The Differential Impacts of Episodic, Chronic, and Cumulative Physical Bullying and Cyberbullying: The Effects of Victimization on the School Experiences, Social Support, and Mental Health of Rural Adolescents. Smokowski, P. (2014)	Violence Victims	54
Loneliness, parent-child communication and cyberbullying victimization among Spanish youths. Larranaga, E. (2016)	Comput Hum Behav	52
Cyberbullying among college students with disabilities. Kowalski, R. (2016)	Comput Hum Behav	50
A process model of cyberbullying in adolescence. Lazuras, L. (2013)	Comput Hum Behav	50
Psychosocial Risk Factors for Involvement in Bullying Behaviors: Empirical Comparison Between Cyberbullying and Social Bullying Victims and Bullies. Navarro, R. (2015)	Sch Ment Health	48

Cites (Times cited in Web of Science Core).
