# Supplementary figures and images for: Science Mapping: A Bibliometric Analysis on Cyberbullying and the Psychological Dimensions of the Self
Source: Int J Environ Res Public Health. 2022 Dec 23;20(1):209. doi: 10.3390/ijerph20010209 (PMC9819207; doi:10.3390/ijerph20010209)

Figure S3. Graph with 48 documents most cited and links between them.

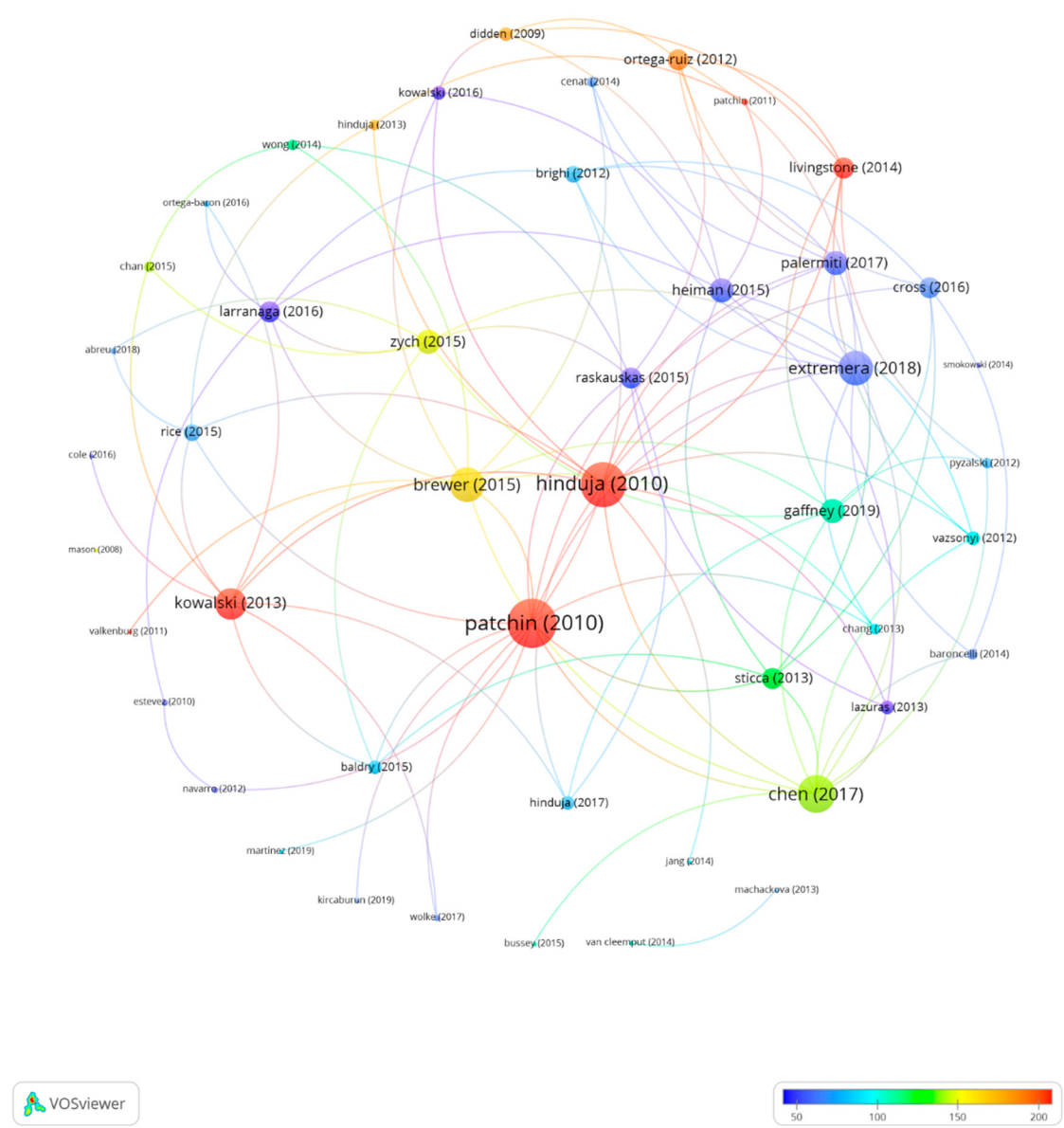

Supplement: Supplementary file 1 [file ijerph-20-00209-s001.zip › Figure S3. Graph with 48 documents most cited and links betweens them.pdf]

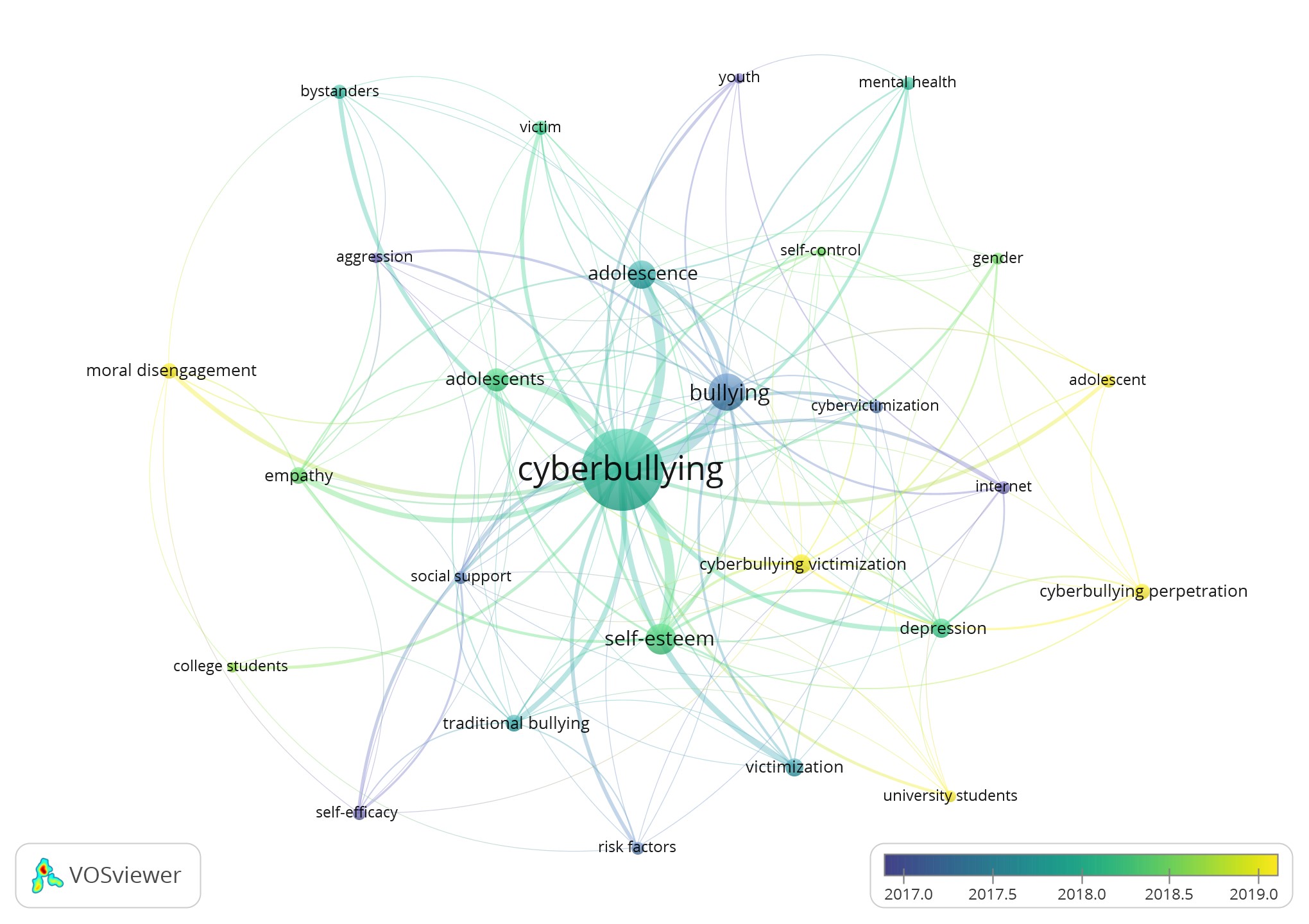

Supplement: Supplementary file 1 [file ijerph-20-00209-s001.zip › Figure S4. Keywords.jpg]
